# Laparoscopic Sacral Colpopexy: A Proposed Technique

**DOI:** 10.1155/DTE.2.43

**Published:** 1995

**Authors:** Cynthia C. Goldberg, Joel M. Childers, Earl A. Surwit

**Affiliations:** Department of Obstetrics and Gynecology University of Arizona 2625 N. Craycroft Road Tucson AZ 85724 USA

## Abstract

This case report describes a laparoscopic sacral colpopexy using Mersilene mesh in a patient with complete vaginal vault prolapse. Mersilene mesh was placed as a hammock between the vaginal apex and the anterior surface of the sacrum, using intracorporeal needles and an extracorporeal knot tying technique. Minor modifications are made from the traditional abdominal approach, because the patient had previously undergone a pelvic lymphadenectomy and vaginal cuff radiation for a stage IB grade 1 adenocarcinoma of the endometrium.


*Diagnostic and Therapeutic Endoscopy*, 1995, Vol. 2, pp. 43–46
Reprints available directly from the publisher
Photocopying permitted by license only

© 1995 Harwood Academic Publishers GmbH
Printed in Singapore

### Laparoscopic Sacral Colpopexy: A Proposed Technique

CYNTHIA C. GOLDBERG, JOEL M. CHILDERS and EARL A. SURWIT

*Department of Obstetrics and Gynecology, University of Arizona*

*(Received September 9, 1994; in final form February 27, 1995)*

This case report describes a laparoscopic sacral colpopexy using Mersilene mesh in a patient with complete vaginal vault prolapse. Mersilene mesh was placed as a hammock between the vaginal apex and the anterior surface of the sacrum, using intracorporeal needles and an extracorporeal knot tying technique. Minor modifications are made from the traditional abdominal approach, because the patient had previously undergone a pelvic lymphadenectomy and vaginal cuff radiation for a stage IB grade 1 adenocarcinoma of the endometrium.

**KEY WORDS:** laparoscopy, Mersilene mesh, sacral colpopexy, vaginal vault prolapse,

### INTRODUCTION

Vaginal surgeons will be faced with an increasing number of patients in which surgical correction of vaginal vault prolapse is necessary. This is largely due to the continually enlarging geriatric population and compounded by the large numbers of women who have had a hysterectomy. Both aging and inadequate support of the vaginal vault at the time of hysterectomy contribute to vaginal vault prolapse (1). There are a variety of procedures available to correct this problem. In the patient who desires preservation of a coitally functional vagina, an abdominal sacral colpopexy with retroperitoneal interposition of a synthetic suspensory hammock between the vaginal apex and the anterior surface of the sacrum can be used. Sacral colpopexy as first described in 1973, and subsequent modifications, require a midline abdominal incision and the accompanying hospitalization and recovery time associated with a laparotomy (2). We report the use of operative laparoscopy for this procedure to circumvent these disadvantages of laparotomy.

### Case Report

Our patient is a 69-year-old, gravida four, para three white female, with a history of stage IB grade endometrial cancer. Her malignancy was managed with a laparoscopically assisted vaginal hysterectomy and pelvic lymphadenectomy, and anterior and posterior colporrhaphy for prolapse. This was followed by high dose vaginal cuff radiation, with 1500 cGy administered in 3 fractions. Approximately two years post-operatively, the patient presented complaining of protrusion of the vaginal vault and pelvic pressure. An examination revealed complete vaginal vault prolapse without an accompaning cystocele or rectocele. She was initially treated with a donut pessary which the patient was not satisfied with so surgical correction was undertaken.

The procedure was performed with the patient in the dorsal lithotomy position and the bladder drained. A 10 mm umbilical port, two 5 mm lateral lower abdominal ports, and one 12 mm suprapubic port were used. The vagina was elevated by a sponge on a ring forceps in the vaginal vault. A Moschcowitz culdoplasty was performed to obliterate the deep cul-de-sac (Fig. 1). Three circumferential purse string sutures included the remnants of uterosacral ligaments, the posterior vagina, and peritoneum, and shallow bites of serosa over the sigmoid-colon laterally. This was performed using 000 coated nonabsorbable braided Ethibond sutures (Ethicon, Sommerville, MA) on a CT needle. Knots were tied extracorporeally using the Clarke's knot pusher.

The Mersilene mesh (Ethicon, Sommerville, MA) was then placed through the 12 mm port. With the index and middle fingers in the vagina, the vaginal apex was elevated. Three Ethibond sutures were placed in a single row in the vaginal wall apex, and then through one end of the

Address for correspondence: Joel Childers M.D., 2625 N. Craycroft Road, Tucson, AZ 85724, 520-324-2469.

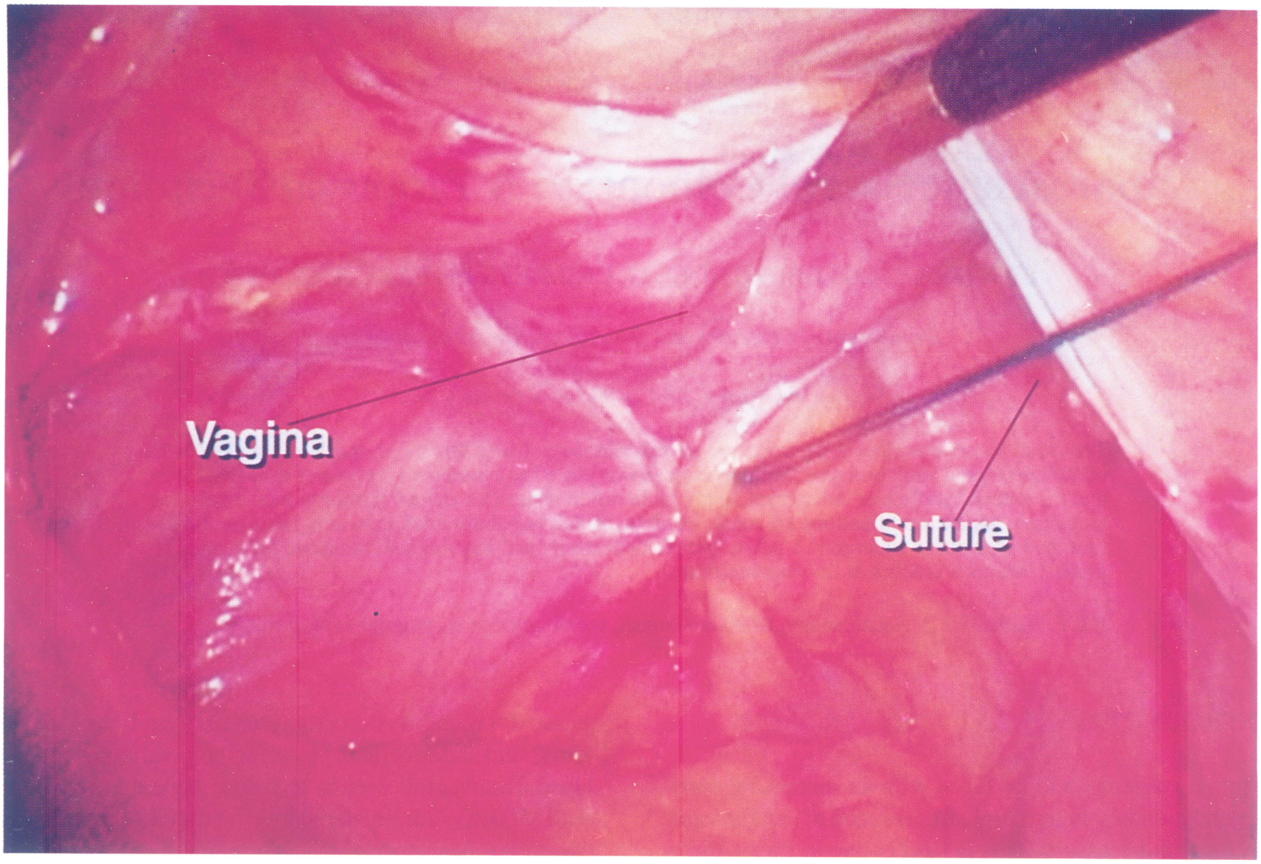

**Figure 1** The first circumferential row of purse-string sutures has been placed and secured using an extra corporeal knot tying technique. The vagina is elevated to avoid being closed behind the Moschcowitz culdoplasty.

Mersilene mesh, and tied using an extracorporeal knot tying technique. By palpating the needle with the fingers in the vagina, good linear bites of the vagina could be obtained without entering the vagina with the suture (Fig. 2).

The parietal peritoneum overlying the promontory of the sacrum was then sharply entered on the right side of the mesentery of the rectosigmoid. The right ureter was identified, and the sacral promontory was cleaned free of its peritoneal and lymph vascular tissue. The periosteum over the sacral promontory was used as an anchoring point. Three sutures were placed anchoring the Mersilene gauze to the sacral promontory using O Ethibond suture. The redundant Mersilene gauze was cut and extracted through the suprapubic port. The gauze bridge was then peritonealized by closing the peritoneum of the sacral promontory, using the clip applier through the 12 mm port (Fig. 3). The vagina was then packed for 24 hours. The operative time was one hour and fifty-two minutes, with an estimated blood loss of 50 ml.

On post-operative day number one, the foley catheter was removed and the patient was discharged home with stool softeners to prevent constipation and associated straining. On examination six weeks postoperatively, the vaginal apex was well supported centrally. Twelve months postoperatively, the patient is asymptomatic and sexually active without problems.

### DISCUSSION

Abdominal colpopexy by suspending a mesh hammock between the prolapsed vaginal vault and sacrum is an accepted surgical technique with good results in the repair of vaginal vault prolapse. This method has been chosen for patients in whom a coitally functional vagina is paramount. Addison described 54 of 56 patients, followed on average 3 years after abdominal sacral colpopexy, to have exhibited good vault suspension and no difficulty with coitus (3). Timmons described 163 patients, of those, 99%

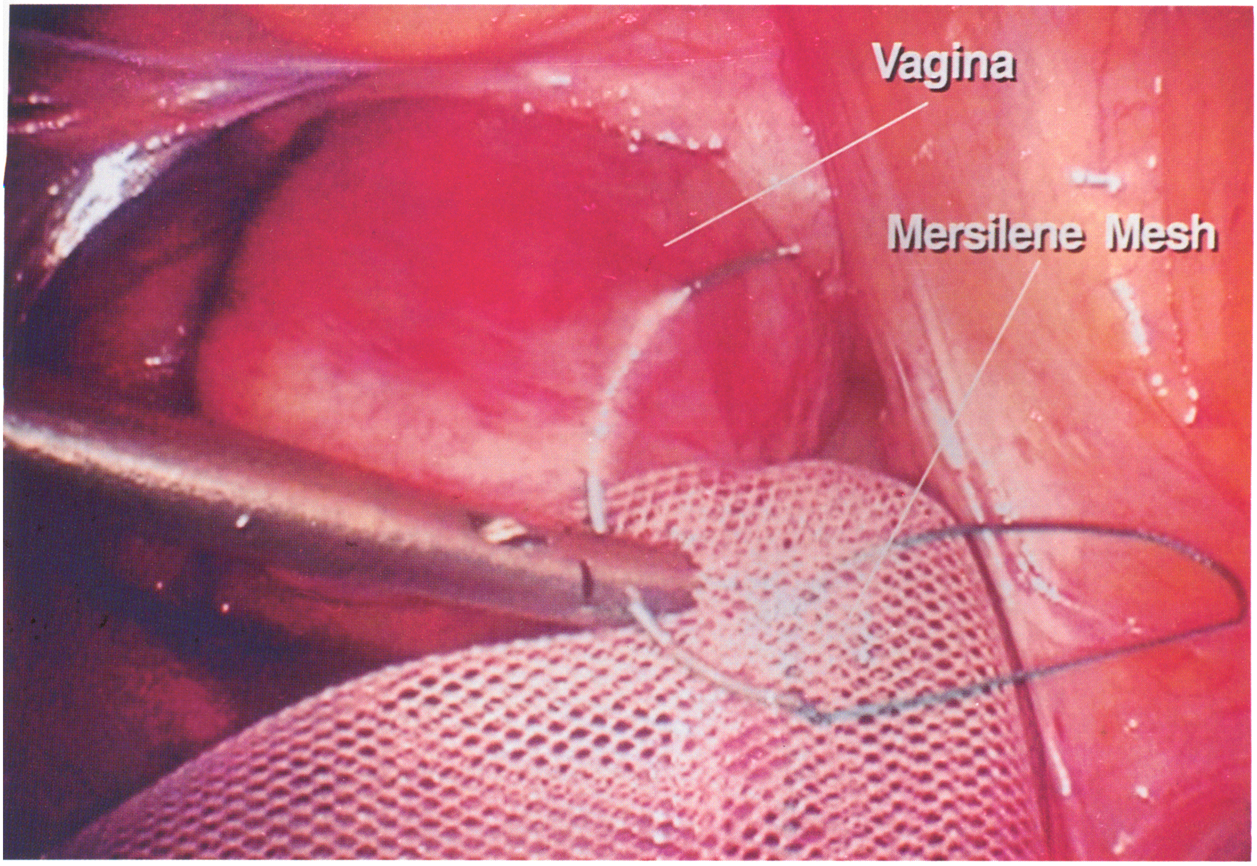

**Figure 2** After placing the suture through the mesh, the surgeon secures an ample portion of vaginal tissue, by driving the needle with his/her right hand and elevating the vagina, palpating the needle with his/her left fingers.

had good support at 9–18 months follow-up with only 2% complicated by recurrent enteroceles (4).

Variations in the surgical procedure have been reported by numerous authors. The Halban technique to vertically close the cul-de-sac has been used in place of the Moschcowitz culdoplasty (5). Different bridge materials and techniques to attach the bridge to the vagina have been reported (3,4). (Timmons et al, 1992). Whether the hollow of the sacrum or the sacral promontory should be used as the fixation point and whether simultaneous prophylactic urethropexy should be performed are debated issues.

In our patient, the bladder and rectum were not near the previously radiated vaginal apex, therefore dissection of the peritoneum off the vaginal mucosa was not necessary. We attached the graft to the anterior sacrum, not the hollow of the sacrum because of its easier accessibility, minimal venous plexous and better periosteum for anchoring sutures. Short-term follow up of this patient has demonstrated this angle to be without consequence. The lateral angle created by the sacrospinal ligament fixation, while not entirely natural, is without problems as well (6).

There are multiple benefits of a minimally invasive laparoscopic approach. The obvious benefits include a smaller incision, decreased blood loss and decreased hospitalization and recovery time. Theoretically, there should be an overall decrease in the complications associated with a laparotomy, such as wound infection and dehiscence, deep venous thrombophlebitis, adynamic ileus, and prolonged recovery.

We have described a surgical technique for laparoscopic sacral colpopexy using Mersilene mesh and its relative advantages. However, a larger series of patients is required before conclusions can be reached concerning its feasibility, safety, limitations and success rate. Nezhat has reported 5 cases of laparoscopic sacral colpopexy, but has also been unable to present the long-term benefits (7). Initial experience by ourselves and others indicate that in properly selected patients this technique is worthy of further investigation.

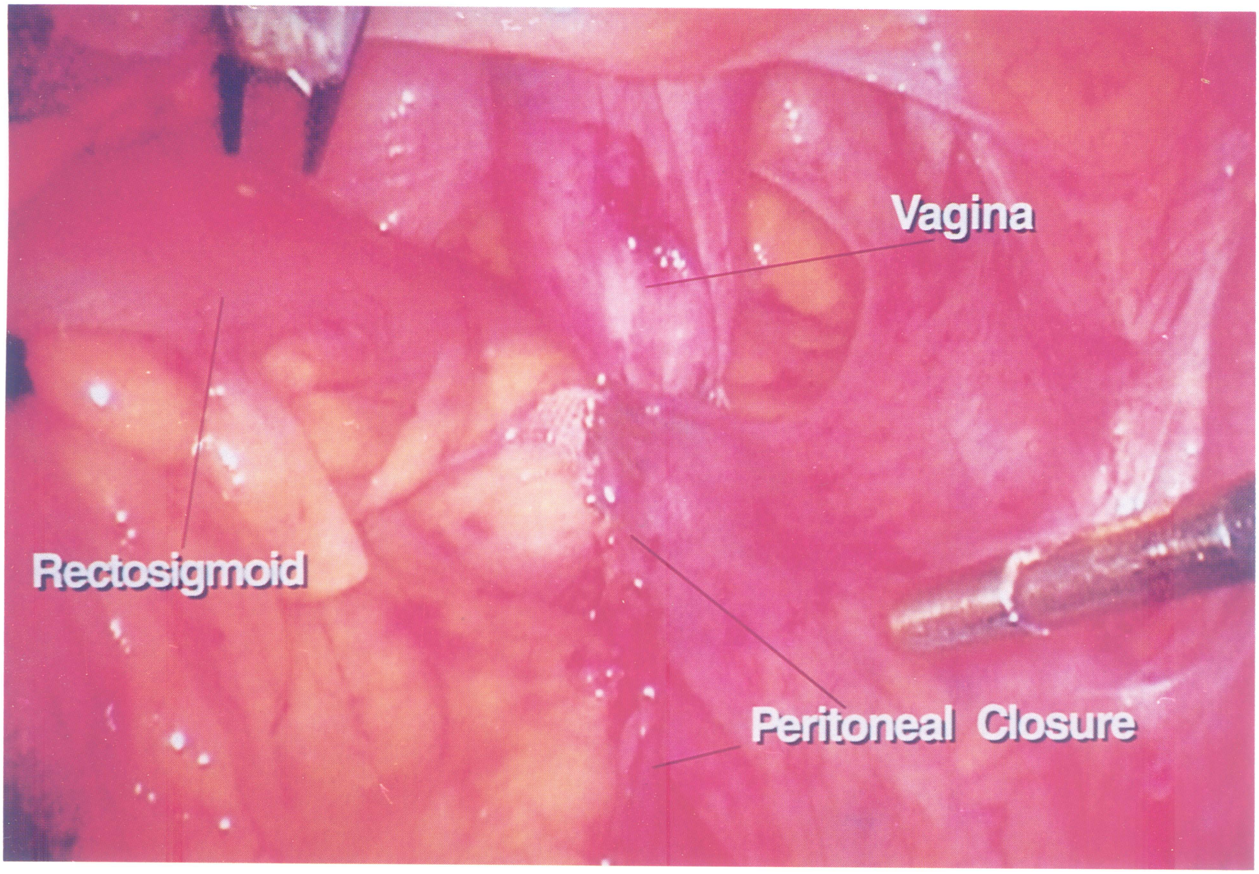

**Figure 3** The Mersilene mesh has been "reperitonealized" to avoid its exposure to intraperitoneal structures.

### REFERENCES

1. Hendee AE. Sacral colpopexy for enterocele and vaginal vault prolapse. In: Thompson JD, Rock JA. (eds) Te Linde's Operative Gynecology, Seventh Edition. J.P. Lippincott, Philadelphia, 1992.
2. Birnbaum SJ. Rational therapy for the prolapsed vagina. Am J Obstet Gynecol, 1973;115:411.
3. Addison WA, Livengood CH, et al. Abdominal sacral colpopexy with Mersilene mesh in the retroperitoneal position in the management of post-hysterectomy vaginal vault prolapse and enterocele. Obstet Gynecol, 1985;153:140.
4. Timmons MC, Addison WA, et al. Abdominal sacral colpopexy in 163 women with post-hysterectomy vaginal vault prolapse and enterocele, evolution of operative techniques. J Reprod Med 1992; 37:323.
5. Nichols DH, Randall CL. Vaginal Surgery. 3rd Edition. Baltimore, MD. Williams & Wilkins. 1989;322–323.
6. Nichols DH. Enterocele and Massive Eversion of the Vagina. In: Thompson JD, Rock JA. (eds) Te Linde's Operative Gynecology, Seventh Edition. J.P. Lippincott, Philadelphia, 1992.
7. Nezhat C, Nezhat F, Nezhat C: Operative laparoscopy (Minimally invasive surgery): State of the Art. J Gyn Surg, 1992;8:111.

